# Comparison of 10 single and stepped methods to identify frail older persons in primary care: diagnostic and prognostic accuracy

**DOI:** 10.1186/s12875-016-0487-y

**Published:** 2016-08-03

**Authors:** Fleur L. Sutorius, Emiel O. Hoogendijk, Bernard A. H. Prins, Hein P. J. van Hout

**Affiliations:** 1 Department of General Practice and Elderly Care Medicine, EMGO+ Institute for Health and Care Research, VU University Medical Center, Van der Boechorststraat 7, Amsterdam, 1081 BT The Netherlands; 2 Department of Epidemiology & Biostatistics, EMGO+ Institute for Health and Care Research, VU University Medical Center, Amsterdam, The Netherlands; 3 Associated General Practitioners Amsterdam Groot-Zuid, Amsterdam, Netherlands

**Keywords:** Frail elderly, Accuracy, Frailty identification, Stepped approach, Primary care, Older people

## Abstract

**Background:**

Many instruments have been developed to identify frail older adults in primary care. A direct comparison of the accuracy and prevalence of identification methods is rare and most studies ignore the stepped selection typically employed in routine care practice. Also it is unclear whether the various methods select persons with different characteristics. We aimed to estimate the accuracy of 10 single and stepped methods to identify frailty in older adults and to predict adverse health outcomes. In addition, the methods were compared on their prevalence of the identified frail persons and on the characteristics of persons identified.

**Methods:**

The Groningen Frailty Indicator (GFI), the PRISMA-7, polypharmacy, the clinical judgment of the general practitioner (GP), the self-rated health of the older adult, the Edmonton Frail Scale (EFS), the Identification Seniors At Risk Primary Care (ISAR PC), the Frailty Index (FI), the InterRAI screener and gait speed were compared to three measures: two reference standards (the clinical judgment of a multidisciplinary expert panel and Fried’s frailty criteria) and 6-years mortality or long term care admission. Data were used from the Dutch Identification of Frail Elderly Study, consisting of 102 people aged 65 and over from a primary care practice in Amsterdam. Frail older adults were oversampled. The accuracy of each instrument and several stepped strategies was estimated by calculating the area under the ROC-curve.

**Results:**

Prevalence rates of frailty ranged from 14.8 to 52.9 %. The accuracy for recommended cut off values ranged from poor (AUC = 0.556 ISAR-PC) to good (AUC = 0.865 gait speed). PRISMA-7 performed best over two reference standards, GP predicted adversities best. Stepped strategies resulted in lower prevalence rates and accuracy. Persons selected by the different instruments varied greatly in age, IADL dependency, receiving homecare and mood.

**Conclusion:**

We found huge differences between methods to identify frail persons in prevalence, accuracy and in characteristics of persons they select. A necessary next step is to find out which frail persons can benefit from intervention before case finding programs are implemented. Further evidence is needed to guide this emerging clinical field.

**Electronic supplementary material:**

The online version of this article (doi:10.1186/s12875-016-0487-y) contains supplementary material, which is available to authorized users.

## Background

The growing number of frail elderly present a major challenge for primary health care regarding timely identification and delivery of adequate care [1, 2]. Dependent on the definition used for frailty and the method of selection between 4 to 59.1 % of people aged 65 years or older are considered to be ‘frail’ [3]. A frequently used definition of physical frailty was defined first by Fried and echoed by Morley et al. in a consensus group [4, 5]. In addition broader definitions of frailty were proposed that also account for psychosocial issues [6]. Both approaches well recognise that frail individuals are highly vulnerable to adverse health outcomes, such as dependency, disability, need for long term care and death [7]. Timely identification of this group of elderly at risk of adverse health outcomes, followed by targeted intervention may promote self-reliance and prevent or delay functional decline [8].

Primary care practices are well positioned to identify frail persons [9]. Primary care is currently going through a transition, changing from a reactive demand-driven approach, in which care is delivered in response to the patients’ complaints and expectations, toward a proactive population-based approach in which it is the professional’s task to actively identify persons at risk and act upon this. One of the prerequisites for successful proactive care for frail elderly, is to obtain a clear overview of frail elderly in the general practice [10].

Identification and selection of frail elderly in primary health care may take place in different ways and from different perspectives. Selection may be based on the general practitioner’s (GP) judgment, the use of available information from electronic medical records, objective risk scores by professionals or self-report. Also, although rarely used, the perspective of elderly themselves may be used to identify frail persons [11–13]. To enable reliable, valid and feasible detection of frail elderly by GPs there is a need for a simple non-time-consuming instrument selecting frail persons at high risk for adverse outcomes who may benefit from a geriatric care intervention [13, 14].

To better target the efforts of professionals and limit burden for elderly it may be favourable to use a stepped approach. For example, prior knowledge available in the general practice may be used to make a first preselection on possible frail older adults. This preselection may be based on their own judgement or information from electronic medical records (EMR) such as polypharmacy or multimorbidity, after which a specific selection method can be used for identification of frailty. Previous studies ignore this stepped practice, typical for primary care, and its consequences for accuracy and prevalence is unknown.

Moreover, it is unknown to which extent the various methods select persons with different characteristics.

Hoogendijk et al. [12] tested the accuracy of 5 easy-to-use instruments to identify frail elderly in primary care. They reported on recommended cut-offs only, and did not study on stepped approaches typical for primary care. In our study we compared 5 additional instruments to identify frail older adults. In addition we analysed the performance of different cut off values as well as stepped strategies. Previous studies have evaluated the validity of several instruments and their ability to predict adverse health outcomes. However a direct comparison of multiple identification methods let alone a stepped approach for identifying frail older adults in primary care does not yet exist. Neither has there been a direct comparison of their prediction of adverse outcomes over 6 years [4, 5, 15–25]. Finally, as prevalence of current instruments vary considerably, they are likely to select persons with different characteristics. Previous studies were unable to compare instruments on such characteristics.

The aim of this study was to estimate the accuracy of 10 different (stepped) methods to identify frailty in older adults in primary care to predict adverse health outcomes. In addition, the methods were compared on their prevalence of identified frail persons and on the characteristics of persons identified.

## Methods

### Design and study sample

Ten methods and stepped strategies were compared against two reference standards as well as their prediction of adverse outcomes over 6 years. Data were used from the Identification of Frail elderly Study in the Netherlands. The Identification of frail elderly study concerned a pilot study for a large trial All patients aged 65 and over from a primary care practice in Amsterdam (*n* = 606) received, together with a postal invitation for the annual influenza vaccination, a short questionnaire, including the Groningen frailty indicator (GFI). A total of 63 % of the patients returned the questionnaire (*n* = 383) of whom 256 were willing to participate in a comprehensive assessment. Age and sex did not differ significantly between responders and non-responders. 120 patients were selected from this group, stratified by sex and GFI score. Based on GFI score, patients were divided into three different groups: non-frail (GFI < 2), some frailty (GFI 2-3), moderate to severe frailty (GFI ≥4). Frail older adults were oversampled to ensure inclusion of sufficient frail individuals. In this way we achieved to compose a small but rich and efficient sample. Selected patients were subsequently approached for a comprehensive assessment that included all identification methods.

**Fig. 1:**
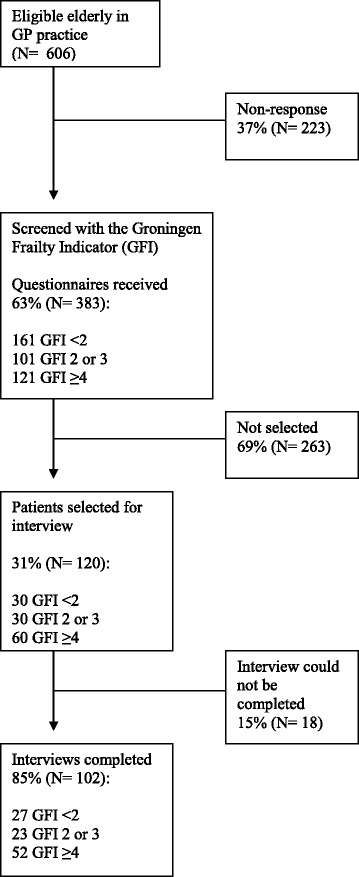
Flowchart of the study population

Data were collected by trained interviewers (medical students and geriatric nurses) between October 2009 and December 2009 by means of computer-assisted personal interviewing and performance tests. In addition, 6 year follow up data between Autumn 2009 and September 2015 on mortality and long term care admission were collected prospectively. The final data set consisted of 102 respondents, some patients refused to participate, others were unable to complete the interview. Figure 1 displays the participants’ flow. Adverse outcomes, mortality and long term care admissions over 6 years, were collected by checking electronic medical files. Only three persons could not be traced because they had moved. The Medical ethics committee of the VU University Medical Center approved the study. Signed informed consent was obtained by all study participants [12].

In addition to the clinical judgement of the GP, polypharmacy, PRISMA-7, the Groningen Frailty Indicator (GFI) and self-rated health of the patient [12], we compared the Frailty Index (FI) and the Edmonton Frail scale (EFS) and 3 other tools used to identify older adults at risk for adverse health outcomes: Gait speed, the Identification Seniors At Risk Primary Care (ISAR PC) and the InterRAI screener. Although not all instruments are designed to identify frailty specifically, target groups are similar to that of the frailty screening instruments (older adults at risk for adverse health outcomes) which makes comparison interesting. Complete lists of all instruments and corresponding items can be found in the Additional file 1.

### Index tests: selection methods

#### Clinical judgment of the GP

The GP made a clinical judgment about each patient. By answering the question: ‘Would you consider this patient to be frail, if frailty is defined as a loss of resources in several domains of functioning (physical, psychological, social), increasing the risk of adverse outcomes?’

#### GFI

A 15-item screening instrument, measuring loss of functioning and resources in physical (9 items), cognitive (1 item), psychological (2 items) and social domain (3 items). A summed score of 4 or more is considered to indicate frailty [15].

#### PRISMA-7

A brief 7-item questionnaire form to identify considerably disabled older adults in order to prevent or delay functional decline. It has previously been used in frailty studies. A score of ≥3 is considered to identify frailty. Questions cover gender, autonomy, walking, social support and environment [21].

#### FI

The FI calculates the proportion of potential deficits present in an individual (such as symptoms, signs, diseases and disabilities). It consists of a minimum of 30 items, associated with ageing and health status. The index is expressed as a ratio of deficits present to the total number of deficits considered ranging from 0 to 1, a score of 0.25 indicates frailty. We constructed a Frailty Index from available data collected during the interviews, following a standard procedure described by Searle et al. [20]. Variables satisfying the set criteria were included in the FI and can be found in the Additional file 1.

#### EFS

A brief and reliable tool which can be used by clinicians without special training to assess the frailty of the older patient. It assesses 10 domains including cognition, health status, functional reliance, social support, medication, nutrition, mood, continence, balance and mobility. Each item is scored, ≥4 indicates slight frailty, ≥6 moderate frailty, a maximum of 17 point indicates the highest level of frailty. Previous research indicated that the EFS had good agreement level with clinical judgment of geriatricians on frailty. To operationalise the EFS comparable items were selected from previously gathered information during the interviews [18].

#### ISAR PC

An instrument to identify persons aged 75 years and older at increased risk of functional decline in the open population. It comprises three self-report questions on age, dependence in instrumental activities of daily living, and impaired memory. The ISAR-PC is easily applicable and validated in general practice [19].

#### The InterRAI screener

The self-reliance algorithm of the interRAI screener is used in several countries to select people at risk for adverse outcomes. It is derived from The InterRAI Home Care Assessment System, a comprehensive geriatric assessment for home-dwelling community dwelling persons. It consists of 8 items on self-reliance in domains of instrumental Activities of Daily Living, cognition, and general health status. The patient is considered to be frail if one or more items are score as not self-reliant [22].

#### Gait speed

Gait speed was validated for predicting adverse health outcomes and has shown to have high diagnostic value for monitoring frailty in community-dwelling older people [26]. Detection of walking speed <0.8 m/s is a simple approach to the diagnosis of frailty in the primary care setting [23]. The gait speed test was performed on a walkway that was at least 4.57 m (100 inch) long to prevent slowing down. Participants walked on the walkway twice at a regular pace. Gait speed was recorded over 4 m. For our analysis we used the best performance of the two measurements.

#### Polypharmacy

The number of medicine prescriptions was derived from EMR. Different cut-off points of medications with different Anatomic Therapeutic Chemical classification system (ATC) codes prescribed over the past 6 months were applied, indicating moderate to major polypharmacy. There is evidence that polypharmacy is associated with increased risk of mortality in elderly people [24].

#### Self-rated health

Self-rated health of the patient was assessed with the question ‘How would you rate your health status on a scale from 0 to 10?’ Different cut-off points were applied to compare with reference standards. Self-assessed health has shown to be predictive of functional decline and mortality [25].

### Cross sectional and prognostic reference standards

#### Expert panel’s opinion

Eight clinical experts, constituted two expert panels. Each consisted of a GP, a nursing home physician, a geriatrician and a geriatric nurse. Each panel was asked to give their judgment of 51 patient descriptions, which were sent to the panel members by e-mail. Patient descriptions were presented to the panel, following the RAND procedure [27]. Descriptions contained general demographic information, MMSE score, functional and psychological information from the InterRAI-CHA [28] and medical history. Members of the panel rated each patient on the CSHA 7-point Clinical frailty scale [7]. Frailty was defined as a score of 5 or higher. In the case of disagreement between panel members on the judgment, members were asked to reconsider the score and after consesus was reached a final classification ‘frail’ or ‘not frail’ was given.

#### Fried’s frailty criteria

Measures 5 physiological items: unintended weight loss, self-reported exhaustion, weak grip strength, slow walking speed and reduced physical activity. A person was considered frail if 3 or more criteria were present [5]. In the USA these criteria are considered as gold standard for measuring physical frailty.

#### Adverse outcome

Mortality or long term care admission over 6 years (Autumn 2009-September 2015) were collected by checking electronic medical records of the GP. Long term care was defined as services provided by nursing homes and assisted living facilities for persons who were unable to manage independently in the community.

### Additional variables

In addition to the index and reference standards a comprehensive geriatric assessment was performed comprising past education, working experience, the Community Health Assessment version of the Resident Assessment Instrument (RAI-CHA) supplemented with MMSE, clock-drawing test, and performance tests (grip strength, walking speed and physical activity). Additional variables were used for comparison of characteristics or to make a preselection in the stepped approaches. International Classification of Primary Care (ICPC)-codes were derived from EMR to determine the number of chronic diseases. Receiving homecare consisted of receiving homemaking (such as cleaning, yard work, laundry), meal preparation services, personal care (help with bathing, getting dressed, eating, use of toilet), supporting assistance (walking, finance, administration and nursing, administration of medication, wound care, catheterisation). Low MMSE-score was defined as one standard deviation below Dutch age and educational population norms. Furthermore patients were characterised as being without partner, having one or more IADL dependencies, self-reported sadness and age older than 80 years.

### Statistical analysis

For statistical analysis we made use of SPSS 22.0 (SPSS Inc. Chicago, Illinois).

To estimate the test accuracy of the instruments compared to both the cross sectional and prognostic reference standards we determined the Area under the Receiver Operating Characteristic (ROC) curve, also known as the AUC-index. The AUC values range from 0.5 to 1.0. An area of 1.0 represents a perfect test, meaning perfect sensitivity and specificity. 0.90–1.0 = excellent; 0.80–0.90 = good; 0.70–0.80 = fair; 0.60–0.70 = poor; 0.50–0.60 = fail [28].

As the ISAR PC was developed for people aged 75 years and over, we determined AUC-values for the ISAR PC for both people aged 75 years and 65 years and older.

Prevalence of frailty according to the different identification methods was determined and expressed by proportions. In case of continuous index tests, performance of different cut-offs values was assessed. Prevalence and diagnostic accuracy were also calculated for several stepped approaches. Pre-selections were made using ready available data in the general practice (number of prescribed medicine, number of diseases and judgment of GP). Finally, we compared prevalence of characteristics (aged 80 years and over, iADL dependency, receiving homecare, low MMSE-score, living without a partner) of the frail persons identified by the various methods. Chosen characteristics are global indicators of vulnerability typically related to frailty and easy to recognise by practitioners. Moreover a recent large trial demonstrated beneficial impact of geriatric primary care if frail persons had additional vulnerability characteristics [29].

We used Cohen’s Kappa to calculate the level of agreement between the instruments and the cross sectional reference standards. Values between 0.60 and 1.0 indicate substantial to almost perfect agreement.

### Weighing

Because of oversampling of frail persons and the stratified selection, outcomes were weighted back to the GFI composition of the population that had returned the postal GFI questionnaire (*n* = 379), from which the selection of 102 respondents was made to estimate prevalence representative for the primary care practice [12].

## Results

Table 1 shows demographic and health characteristics of included participants.

**Table 1:** Participants’ demographic and health characteristics (*n* = 102)

Age, 65–96 mean (SD)	78.6 (7.1)
Sex, % women	56.9
Educational Level, 1–8, %	
Low (1–2)	10.3
Middle (3–6)	41.2
High (7–8)	48.5
Lives alone %	48
Body Mass Index, 0–37 mean (SD)^a^	24.8 (7.2)
Underweight %	0
normal %	34.4
overweight %	47.9
severe overweight %	17.7
Number of prescribed medicine, mean (SD)	4.1 (3.2)
Number of chronic diseases, mean (SD)	2.9 (1.9)
Self-rated health, 0–10 mean (SD)	6.5 (1.5)
MMSE score, 0–30 mean (SD)	26.9 (2.2)
Dependency in mobility,0–4^b^, mean (SD)	0.3 (0.6)
Toilet use %	0
Groceries %	17.0
Walking %	8
Dressing %	3.9

^a^Body mass index: <18.5 underweight; 18.5–24.99 normal weight; 25–29.99 overweight; ≥30 severe overweight

^b^Based on 4 GFI items on mobility. Each item scored dependent (0) or independent (1). Use of helping devices is considered independent

### Prevalence and cross sectional accuracy of identification methods

**Fig. 2:**
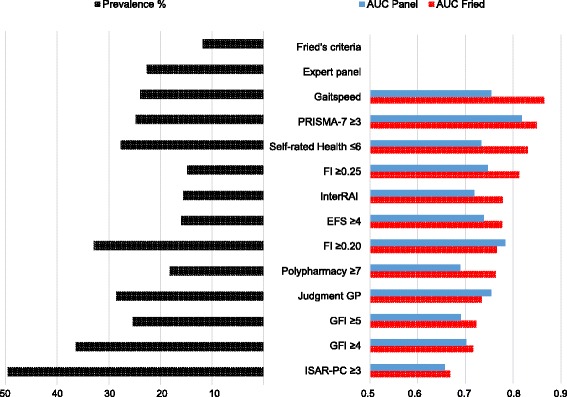
Prevalence and cross sectional Accuracy of identification methods

Figure 2 shows the prevalence and Area under the curve (AUC) compared to Fried’s criteria and the expert panel’s opinion as reference standard. The results displayed are for identification method’s cut offs that had the highest AUC-values for both reference standards. For the FI and GFI there was no consistency on highest AUC between reference standards for different cut offs. Both cut offs are therefore displayed in Fig. 2.

A complete summary of outcome measurements for all identification methods including different cut offs can be found in Table 2. Prevalence rates ranged from 14.8 % (FI) and 52.9 % (ISAR PC) between instruments and AUC values ranged from poor (0.635 for ISAR PC compared to the expert panel) to good (0.865 for gait speed compared to Fried’s criteria) for recommended cut off values.

**Table 2:** Prevalence and accuracy and agreement of identification methods (weighted analyses)

Reference standard		Fried’s frailty criteria (Ref.) frail 11.6 %				Expert panel (Ref.) frail 22.8 %			
Identification method	Frail %	AUC	Asymptotic 95 % confidence interval		Kappa fried	AUC	Asymptotic 95 % confidence interval		Kappa panel
			Lower	Upper			Lower	Upper	
PRISMA-7									
≥ 3	24.8	0.849	0.830	0.869	0.469	0.818	0.800	0.836	0.612
≥ 4	11.9	0.723	0.693	0.753	0.442	0.699	0.676	0.722	0.476
≥ 5	3.6	0.594	0.594	0.594	0.270	0.546	0.523	0.569	0.131
Self-Rated Health									
≤ 6	27.7	0.831	0.809	0.853	0.377	0.733	0.712	0.754	0.437
≤ 5	11.1	0.725	0.692	0.659	0.435	0.649	0.625	0.673	0.363
≤ 4	4.6	0.654	0.619	0.689	0.406	0.599	0.575	0.623	0.274
FI									
≥ 0.20	32.9	0.766	0.743	0.789	0.303	0.784	0.765	0.802	0.498
≥ 0.25	14.8	0.813	0.787	0.839	0.566	0.747	0.724	0.769	0.568
≥ 0.30	7.4	0.693	0.662	0.724	0.461	0.644	0.620	0.667	0.376
≥ 0.35	4.9	0.648	0.617	0.680	0.397	0.608	0.584	0.631	0.296
≥ 0.40	1.8	0.578	0.547	0.609	0.244	0.540	0.517	0.563	0.118
Interrai Self-Reliance Screen	15.6	0.778	0.750	0.805	0.483	0.719	0.697	0.741	0.490
EFS									
≥ 4	16.0	0.777	0.750	0.805	0.490	0.738	0.549	0.596	0.199
≥ 6	5.0	0.616	0.585	0.648	0.312	0.573	0.716	0.760	0.534
ATC									
≥ 5	31.9	0.715	0.689	0.741	0.244	0.666	0.645	0.688	0.291
≥ 6	22.5	0.739	0.712	0.766	0.339	0.660	0.638	0.683	0.323
≥ 7	18.2	0.763	0.736	0.790	0.422	0.689	0.666	0.711	0407
Judgment GP	28.6	0.734	0.708	0.760	0.287	0.754	0.734	0.774	0.463
GFI									
≥ 4	36.4	0.716	0.691	0.741	0.225	0.702	0.682	0.723	0.332
≥ 5	25.3	0.723	0.696	0.750	0.296	0.690	0.585	0.631	0.364
≥ 6	12.6	0.633	0.603	0.664	0.257	0.608	0.669	0.712	0.255
ISAR PC									
≥ 2	52.9	0.649	0.624	0.675	0.117	0.635	0.614	0.656	0.185
≥ 3	49.6	0.668	0.643	0.693	0.139	0.657	0.637	0.677	0.223
≥ 4	49.6	0.668	0.643	0.693	0.139	0.657	0.637	0.677	0.223
Gait speed	23.9	0.865	0.846	0.884	0.478	0.754	0.732	0.776	0.490

AUC-values for the GFI compared to Fried’s criteria slightly increased when using a higher cut off of ≥5. A FI cut off of 0.20 instead of 0.25 resulted in a higher AUC when comparing to the expert panel. For the ISAR PC we found a slightly higher AUC for a cut off value of ≥3, instead of ≥2 compared to both Fried’s criteria as well as the expert panel. For all other instruments AUC-values were highest for the recommended cut off values, as proposed by previous research.

Compared to Fried’s criteria the highest AUC was found for gait speed (AUC 0.865). Second highest was PRISMA-7 cut off ≥3. Good discriminative ability was also found for self-rated health (cut off ≤6; AUC: 0.831); and FI (cut off ≥0.25; AUC 0.813). For recommended cut off values the ISAR-PC (≥2) had the lowest AUC (0.649).

When using the expert opinion as reference standard we found similar results. Overall highest AUC was found for PRISMA-7 (Cut of ≥3; AUC 0.818). For recommended cut off values ISAR PC had the lowest AUC score (AUC 0.635). All other cut off values of instruments had an AUC below 0.8 compared to the expert opinion. For participants aged 75 years or older, AUC-values did not increase for the ISAR PC which was developed to for this subgroup (AUC 0.637 and 0.635 compared to Fried resp. expert panel). The highest agreement was found between PRIMSA-7 and the exert panel (к 0.61), followed by FI ≥0.25 and Fried (к 0.57).

### Stepped approach

**Fig. 3:**
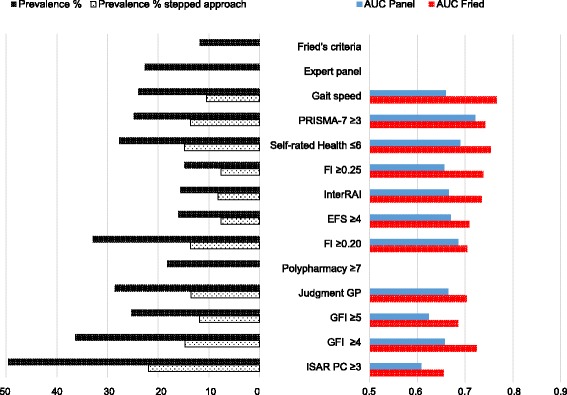
Stepped Approach Accuracy and prevalence for preselection by ATC ≥5

**Fig. 4:**
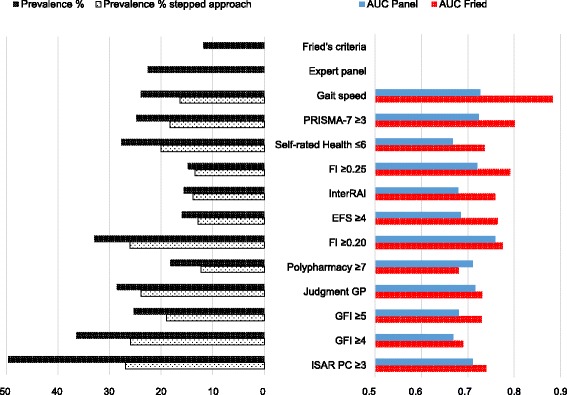
Stepped Approach Accuracy and prevalence for preselection by ICPC ≥2

**Fig. 5:**
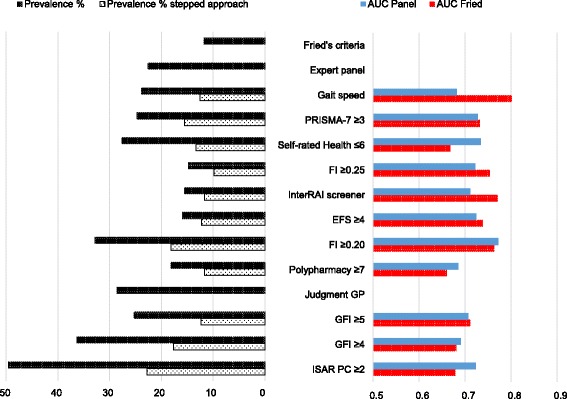
Stepped Approach Accuracy and prevalence for preselection by GP

Preselection on polypharmacy (≥5), ICPC-disease codes (≥2) or the GP’s opinion led to lower prevalence rates for all methods. Figures 3, 4 and 5 show results for respectively preselection by ATC ≥ 5, ICPC ≥ 2 and judgment of the GP. A complete overview of prevalence and accuracy of all stepped approaches can be found in the Additional file 2. Prevalence ranged from 6.4 % (EFS preceded by ATC ≥5) to 30.3 % (ISAR PC preceded by ICPC ≥2) for recommended cut offs. Highest AUC-values are highlighted for every preselection method. In general, AUC-values turned out lower compared to both reference standards, except for slight increases in AUC-values for several stepped strategies with the ISAR PC and the GFI.

The stepped strategy with the highest accuracy turned out to be gait speed preceded by ICPC ≥2 (AUC 0.883 and 0.727), followed by the PRISMA-7 cut off ≥3 preceded by ICPC ≥2 (AUC 0.801 and 0.724) compared to Fried respectively the expert panel. Lowest AUC-values were found for the GFI cut off ≥4 preceded by ICPC ≥4.

### Prognostic accuracy of identification methods

**Fig. 6:**
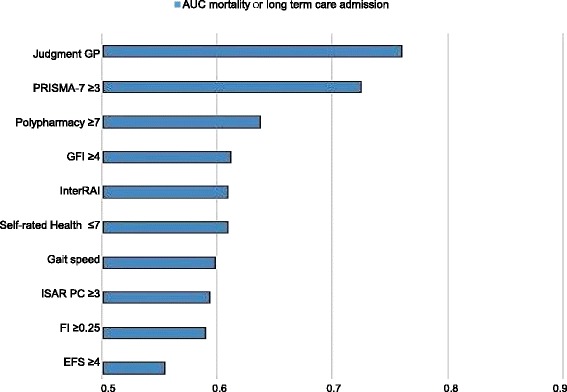
Prognostic Accuracy of Identification methods

Figure 6 illustrates the accuracy of the identification methods to predict mortality (*n* = 18) or long term care admission (*n* = 12). AUCs varied from 0.556 (EFS) to 0.761 (judgement GP).

### Comparison of characteristics of selected persons

Table 3 compares characteristics of participants identified as frail according to various methods using cut offs with the best AUC, including the prevalence of persons scoring at least 1 standard deviation below their age and education specific MMSE-norm score.

**Table 3:** Characteristics of frail older adults

65+ yrs and frail according to	Without partner %	Age >80 %	Home-care^a^ %	IADL-dependency^b^ %	MMSE^c^ %	Self-reported sadness^d^ %
Frieds criteria	33.7	79.8	100	94.8	15.5	20.7
Expert panel	43.7	78.5	92.2	94.6	29.4	21.3
GFI ≥4	31.2	57.6	68.0	60.9	20.0	35.0
Judgment GP	36.3	73.2	79.7	83.3	33.1	20.9
PRISMA-7 ≥ 3	44.5	62.2	79.0	76.4	26.5	22.7
ISAR-PC ≥ 3	51.7	50.6	53.1	91.3	22.0	20.7
FI 0.25	45.9	64.2	82.0	91.7	30.4	38.1
EFS ≥ 4	37.2	61.2	96.1	84.6	39.1	46.7
Self rated health ≤ 6	37.0	58.0	48.2	76.6	19.3	25.9
Polypharmacy ≥ 7	34.7	61.0	70.9	83.5	32.4	26.4
Interrai self-reliance screen	38.9	48.1	82.3	88.5	34.2	30.4
Gait speed	51.9	68.6	70.5	80.3	15.5	10.5
Prevalence range	33.7–51.9	48.1–79.8	48.2–100	60.9–94.8	15.5–39.1	10.5–41
Range ∆	18.2	31.7	51.8	33.9	23.6	30.5

All prevalence rates are weighed

^a^receiving homemaking, personal care, supporting assistance and nursing. ^b^IADL-dependency: needs help with meal preparation, housework, managing finance, using the telephones, walking stairs, shopping, transportation. ^c^mmse-score ≥1 sd under the population norm for age and educational level. ^d^self-reported sadness present in ≤3 days or often present

Among patients selected by both reference standards prevalence of homecare and iADL-dependency was high. Prevalence rates of characteristics between instruments varied widely as is depicted by the prevalence range. This was especially the case for homecare (range 48.2–100 %).

## Discussion

### Main findings

Our aim was to estimate the prevalence and accuracy of different methods and stepped strategies for identifying frail older patients at risk for adverse health outcomes as well as comparing characteristics of identified patients. Prevalence rates varied widely between identification methods, and accuracy ranged from poor to good. Of all investigated identification methods gait speed, the PRISMA-7 questionnaire, self-rated health and the FI achieved the best accuracy compared to the cross sectional reference standards individually as well as used in a stepped strategy. In general, recommended cut off values seem to perform best. Stepped approaches performed worse. The judgment of the GP and PRISMA-7 predicted adverse outcomes best. Instruments selected frail patients who differed greatly in characteristics as age, IADL dependency, receiving home care, and cognitive status.

### Advantages and disadvantages of instruments

The selection instruments we compared used different sources and perspectives: GPs’ opinion; patient opinion; risk scores; physical measurements; available data from the EMR. When using postal questionnaires there is a risk of non-response likely in a biased way. Judgment by the GP is simple and inexpensive, but could be difficult if the GP is not informed well enough about all older patients. Physical measurements are often time-consuming and require visitation. Extraction from EMR avoids the risk of non-response but depends on registration rigour [12].

Next to the PRISMA-7, gait speed and self-rated health, the FI reached a rather high accuracy compared to both reference standards as well. When constructing the FI we extracted variables from the comprehensive assessment, which is not very feasible in practice. However different constructs of FIs have shown to yield comparable results of the risk of adverse outcomes [20]. A FI based on routine health care data (ICPC-codes) of GPs could be a suitable alternative and has shown the ability to predict the risk of adverse health outcomes [11].

We found that the use of pre selection methods could lead to higher efficiency in identifying frail older adults by narrowing down the number of persons needed to evaluate. However, this is accompanied by a loss in accuracy and the risk of missing frail older adults. The benefit of using pre selection methods, especially when using information extracted from the EMR, is to avoid bothering patients and work overload for professionals. Unfortunately registration of ICPC-codes may not always occur accurately, and the GP is not always informed well enough about all patients, therefore pre selection using polypharmacy may be preferable. Further research must follow to determine if pre selection is indeed experienced as beneficial by professionals and is acceptable in number of false negatives.

It should be noted that many instruments include items on disabilities (e.g. PRISMA-7) to measure frailty. Although frailty, disability and comorbidity are inter-related, Fried defines these as distinct clinical entities [30, 31] and integrating disability or comorbidity items into a frailty scale may be debatable. Presence of disabilities on itself may give rise to an increased risk of adverse outcomes [32].

Finally our results suggest that different identification methods select patients with different characteristics. This finding emphasizes the need for consensus on the definition of frailty.

### Strengths and limitations

This study has several strengths. Multiple tools have been developed to identify frailty and have been separately tested and showed reasonable predictive validity [17–26, 33]. This is one of first studies comparing different identification methods directly to both cross sectional and prognostic reference standards. Previous studies comparing instruments did not focus on the population of primary care, included other instruments or did not make use of reference standards [34–36]. Older patients were selected within a general practice, reflecting outcomes for older persons in the general population. Furthermore we evaluated different cut-off values and the use of pre selection methods. Finally, this is the first study to demonstrate heterogeneity across groups selected by different instruments.

A limitation in our study is that data was derived from 102 older adults from just one primary care practice in the Netherlands. The sample contained an above average number of higher educated older adults. Therefore prevalence rates may not depict actual frailty prevalence for the Dutch older population. This is not a restriction for our diagnostic analyses. Furthermore the judgment by the GP on frailty was based on the opinion of only one medical doctor using a pre described definition. Drewes et al. found that judgment by GPs might be a promising instrument, but also found some variability in vulnerability assessment of older adults by different GPs without a prescribed definition [37].

In this study scores for identification methods were derived from available data. Some items for identification methods did not completely mirror the original items such as for the EFS. Next our population focused on persons aged 65 years or older. The ISAR PC is developed for older adults of 75 years or older, however accuracy did not improve when analysis was conducted in this subgroup. Analyses for stepped strategies were only performed in patients 65 years or older.

According to our results gait speed had the highest accuracy against Fried’s criteria. We are aware that gait speed is also one of the five criteria of the fried frailty phenotype which might have inflated it’s accuracy. However, it’s accuracy was also relatively well against the expert panel. A previous study compared gait speed to Fried’s criteria and the FI and found good diagnostic values as well [38]. Moreover, gait speed seems to predict adverse outcomes, and is therefore seen as one of the key indicators of frailty [39, 40].

Some of the characteristics we evaluated are present in the identification instruments as well (e.g. item no.1 of the PRISMA-7 questionnaire is: Are you more than 85 years old?), therefore automatically these identification methods could select patients with these characteristics better than identification methods that do not contain items about corresponding characteristics.

Furthermore, identification methods defined frailty differently. As a gold standard does not exist for measuring frailty we used two different measures of frailty as reference: a one-dimensional, physical concept (Fried’s Phenotype) and a multidimensional concept of frailty (the expert panel). Both have shown to have high predictive ability for adverse health outcomes, making both interesting to use as reference in our aim to identify frail older adults at risk of adverse health outcomes [4, 6].

Finally, frailty detected by most of the identification methods is (highly) associated with adverse health outcomes, however for the PRISMA-7, the judgment of the GP and the InterRAI this relation has not been studied until now.

## Conclusion

We found huge differences between available methods to identify frail persons in prevalence, accuracy as well as in characteristics of the persons they select. A necessary next step is to find out which frail persons can benefit from intervention before case finding programs are implemented. In fact, a recent trial demonstrated beneficial impact of geriatric primary care if frail persons had additional vulnerability characteristics such as being older than 80, living alone and receiving home care [29]. Future studies should test this finding to confirm it’s value and to guide this emerging clinical field. Despite limitations of this study our results could give guidance for general practitioners when choosing a frailty instrument for assessing older adults in their practice. There are numerous instruments available and many instruments are being used currently in practices, yet little is known about how they relate to one another. Our results suggest that several instruments may be suitable for frailty identification. The PRISMA-7, gait speed, InterRAI screener, FI, but also self-reported health could serve as good frailty indicators. Finding a valid identification method for frailty is just the first step, the next step should be a comprehensive evaluation and targeted interventions to modify frailty or to prevent adverse health outcomes such as recently demonstrated by Hoogendijk et al. 2016 [29].

## Abbreviations

ATC, Anatomic Therapeutic Chemical; AUC, the Area Under the Curve; EFS, the Edmonton Frail Scale; EMR, Electronic medical records; FI, the Frailty Index; GFI, the Groningen Frailty Indicator; GP, General Practitioner; ICPC, International Classification of Primary Care; ISAR PC, the Identification Seniors At Risk Primary Care; MMSE, Mini-Mental State Examination; RAI-CHA, the Community Health Assessment version of the Resident Assessment Instrument; ROC, under the Receiver Operating Characteristic.

## Additional files

Additional file 1: Lists of instruments. A complete lists of all instruments and corresponding items. (PDF 741 kb) Additional file 2: Diagnostic test accuracy stepped approaches. A complete overview of prevalence and accuracy of all stepped approaches in table-form. (PDF 154 kb)
