# Supplementary material for: Comparison of 10 single and stepped methods to identify frail older persons in primary care: diagnostic and prognostic accuracy
Source: BMC Fam Pract. 2016 Aug 3;17:102. doi: 10.1186/s12875-016-0487-y (PMC4973108; doi:10.1186/s12875-016-0487-y)
Supplement: Additional file 1: — Lists of instruments. A complete lists of all instruments and corresponding items. (PDF 741 kb) [file 12875_2016_487_MOESM1_ESM.pdf]

Appendix 1

Groningen Frailty Indicator

	YES	NO	SOMETIMES
**Mobility.** Can the patient perform the following tasks without assistance from another person (walking aids such as a can or a wheelchair are allowed)			
1. Grocery shopping	0	1	
2. Walk outside house (around house or to neighbour)	0	1	
3. Getting (un)dressed	0	1	
4. Visiting restroom	0	1	
Vision			
5. Does the patient encounter problems in daily life because of impaired vision?	1	0	
Hearing			
6. Does the patient encounter problems in daily life because of impaired hearing?	1	0	
Nutrition			
7. Has the patient unintentionally lost a lot of weight in the past 6 months (6kg in 6 months or 3kg in 3 months)?	1	0	
Co-morbidity			
8. Does the patient use 4 or more different types of medication?	1	0	
	YES	NO	SOMETIMES
Cognition			
9. Does the patient have any complaints on his/her memory (or diagnosed with dementia)?	1	0	0
Psychosocial			
10. Does the patient ever experience emptiness around him? *e.g. You feel so sad that you have no interest in your surroundings. Or if someone you love no longer love you, how do you feel?*	1	0	1
11. Does the patient ever miss the presence of other people around him? *Or do you miss anyone you love?*	1	0	1
12. Does the patient ever feel left alone? *e.g. You wish there is someone to go with you for something important.*	1	0	1
13. Has the patient been feeling down or depressed lately?	1	0	1
14. Has the patient felt nervous or anxious lately?	1	0	1
Physical Fitness			
15. How would the patient rate his/her own physical fitness? (0-10 ; 0 is very bad, 10 is very good) 0 – 6 = 1 7 – 10 = 0	1	0	
TOTAL SCORE GFI			

Steverink, N., Slaets, J.P.J., Schuurmans, H., & Lis, M. van (2001). Measuring frailty: development and testing of the Groningen Frailty Indicator (GFI). The Gerontologist, 41, special issue 1, 236-237.

PRISMA-7 questionnaire

1. Are you more than 85 years? Yes: 1 No: 0
2. Male? Yes: 1 No: 0
3. In general do you have any health problems that require you to limit your activities? Yes: 1 No: 0
4. Do you need someone to help you on a regular basis? Yes: 1 No: 0
5. In general do you have any health problems that require you to stay at home? Yes: 1 No: 0
6. In case of need, can you count on someone close to you? Yes: 0 No: 1
7. Do you regularly use a stick, walker or wheelchair to get about? Yes: 1 No: 0

Hebert R, Raiche M, Dubois MF, Gueye NR, Dubuc N, Tousignant M. Impact of PRISMA, a coordination-type integrated service delivery system for frail older people in Quebec (Canada): a quasi-experimental study. J Gerontol B Psychol Sci Soc Sci 2010; 65B: 107–18.

List of 38 items used for creating Frailty Index

Bathing performance
Dressing lower Body
Walking performance
Hygiene performance
Stairs performance
Shopping performance
Housework performance
Meal preparation performance
Managing medication performance
Managing finance performance
Weight loss
Self-rated health

Change in social activities
Hours of exercise
Self-reported little interest
Self-reported sadness
lonely
Fatigue
Coronary heart disease
Congestive heart failure
Stroke
Cancer
Diabetes mellitus
COPD
Daily decision making
Short term memory

Dyspneu
Dressing Upper Body
BMI
Pain frequency
Recent Falls
Transportation Performance
Bladder continence
Falls
Dizziness
Unsteady gait
Hearing
Ability to see in adequate light

Protocol of Searle Rockwood Mitniski 2008et al. 2008 "A standard procedure for creating a frailty index" BMC Geriatrics.

Identification Seniors at Risk Primary Care

1. Did you need assistance on a regular basis in the last month (e.g. preparing meals, shopping, housekeeping)?

No = 0.0
Yes = 2.5

2. Do you regularly have memory problems?

No = 0.0
Yes = 2.0

3. What is your age?

74 years or younger = 0.0
Between 75 and 84 years = 1.5
85 years and older = 3.0

Total score …….
Maximum score: 7.5 points
Total score 0 or 1: Not at risk of functional decline
Total score 2 or higher: At risk of functional decline

Suijker J., A simple validated questionnaire predicted functional decline in community-dwelling older persons: prospective cohort studies. J Clin Epidemiol. 2014 Oct;67(10):1121-30.

Edmonton Frail Scale

The Edmonton Frail Scale:				Score:_/17	
**Domain**	**Item**	**0 point**	**1 point**	**2 points**	**Items replaced by:***<br>**(0 Point, 1 Point, 2 Points)**
Cognition	Please imagine that this pre-drawn circle is a clock. I would like you to place the numbers in the correct positions then place the hands to indicate a time of 'ten after eleven'	No errors	Minor spacing errors	Other errors	Cognitive performance Scale (0-1:2-3:1, 4+:2)
General health status	In the past year, how many times have you been admitted to a hospital?	0	1–2	≥2	Hospital admittance in past 90 days no:0 yes:2)
	In general, how would you describe your health?	'Excellent', 'Very good', 'Good'	'Fair'	'Poor'	
Functional independence	With how many of the following activities do you require help? (meal preparation, shopping, transportation, telephone, housekeeping, laundry, managing money, taking medications)	0–1	2–4	5–8	
Social support	When you need help, can you count on someone who is willing and able to meet your needs?	Always	Sometimes	Never	No:0 Yes:1
Medication use	Do you use five or more different prescription medications on a regular basis?	No	Yes		iADL managing medication performance: self reliant:0; needs help:1
	At times, do you forget to take your prescription medications?	No	Yes		
Nutrition	Have you recently lost weight such that your clothing has become looser?	No	Yes		
Mood	Do you often feel sad or depressed?	No	Yes		Depression Rating Scale >2: 1
Continence	Do you have a problem with losing control of urine when you don't want to?	No	Yes		
Functional performance	I would like you to sit in this chair with your back and arms resting. Then, when I say 'GO', please stand up and walk at a safe and comfortable pace to the mark on the floor (approximately 3 m away), return to the chair and sit down'	0–10 s	11–20 s	>20 s patient unwilling, or requires assistance	Locomotion in Home: independent:0, needs limited assistance:1, extensive assistance:2
Final score	The sum of column totals				

* Empty fields indicate original items could be extracted from data

Rolfson DB, Majumdar SR, Tsuyuki RT. Validity and reliability of the Edmonton Frail Scale. Age Ageing 2006; 35: 52

Appendix 6: InterRAI screener

Algorithm:

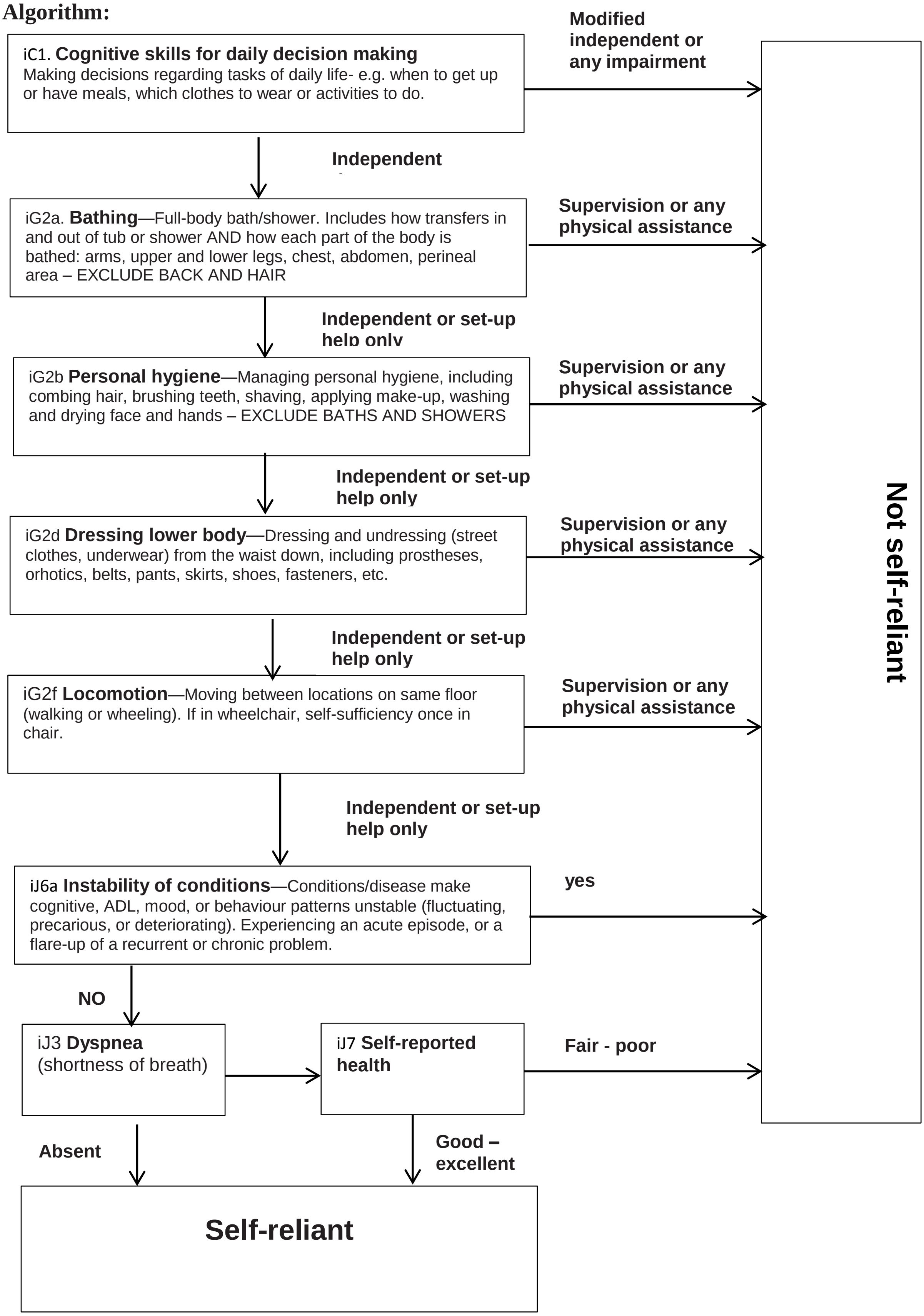

CSHA 7-point Clinical frailty scale

1. Very fit – robust, energetic, well-motivated and fit; these people commonly exercise regularly and are in the most fit group for their age.
2. Well – without active disease, but less fit than people in category 1.
3. Well, with treated comorbid disease – disease symptoms are well controlled compared with those in category 4.
4. Apparently vulnerable – although not frankly dependent, these people commonly complain of being “slow up” or have disease symptoms.
5. Mildly frail – with limited dependence on others for instrumental activities of daily living.
6. Moderately frail – help is needed with both instrumental and non-instrumental activities of daily living,
7. Severely frail – completely dependent om others for the activities of daily living, or terminally ill.

Rockwood K, Song X, MacKnight C, Bergman H, Hogan D. B, McDowell I. A global clinical measure of fitness and frailty in elderly people. CMAJ: Canadian Medical Association Journal. 2005;173(5):489–495..

Frieds Frailty Criteria

Weight loss	In the past 12 months, have you lost more than 10 pounds unintentionally (i.e., not due to dieting or exercise)?
Weakness	Weakness was defined as adjusted mean grip time in the stronger arm in the lowest 20th percentile of a community-dwelling population of adults age 65 and older.<br>Men:<br>BMI≤ 24 kg/m^2: ≤ 29 kg BMI 24.1-26kg/m^2: ≤30 kg<br>BMI 26.1-28 kg/m^2: ≤ 31 kg BMI> 28 kg/m^2: ≤32 kg<br>Women:<br>BMI≤ 23 kg/m^2: ≤ 17 kg BMI 23.1-26kg/m^2: ≤ 17.3 kg<br>BMI 26.1-29 kg/m^2: ≤ 18 kg BMI> 29 kg/m^2: ≤ 21 kg
Exhaustion	Two items from the CES-D*: (1) felt that everything I did was an effort. (2) I could not get "going'. Patients were asked how often in the last week they felt this way, and those who chose "a moderate amount of the time (3-4 days)" or "most or all of the time (5-7 days)" to either question were considered to meet the exhaustion criteria for frailty.
Low activity	Evaluation of 18 activities from the Minnesota Leisure Time Physical Activity Questionnaire. Time spent on each activity is to be recorded in minutes for the last 2 weeks and then multiplied by an activity score.<br>Half of the total of all activities, expressed in kilocalories per week, represents the physical activity of an evaluated individual per week. The physical activity criterion is positive if physical activity per week is<br>for:<br>Male Female<br><383 kcal/week <270 kcal/week
Slow speed walking	Individuals with a walking speed less than the 20th percentile of a community-dwelling elderly population, adjusted for gender and height.<br>Men:<br>Height ≤ 173 cm: ≥ 7 s Height > 173 cm: ≥ 6 s<br>Women:<br>Height ≤ 159 cm: ≥ 7 s Height > 159 cm: ≥ 6 s

*CES-D = Center for Epidemiological Studies Depression.

Fried LP, Tangen CM, Walston J, Newman AB, Hirsch C, Gottdiener J, Seeman T, Tracy R, Kop WJ, Burke g, McBurnie AM. Frailty in Older Adults Evidence for a Phenotype. J Gerontol A, Biol Sci Med Sci. 2001; 56 (3): M146-M
