# Supplementary material for: Comparison of 10 single and stepped methods to identify frail older persons in primary care: diagnostic and prognostic accuracy
Source: BMC Fam Pract. 2016 Aug 3;17:102. doi: 10.1186/s12875-016-0487-y (PMC4973108; doi:10.1186/s12875-016-0487-y)
Supplement: Additional file 2: — Diagnostic test accuracy stepped approaches. A complete overview of prevalence and accuracy of all stepped approaches in table-form. (PDF 154 kb) [file 12875_2016_487_MOESM2_ESM.pdf]

Appendix 9 Diagnostic test accuracy stepped approaches

PRESELECTION ATC ≥ 5 (31.9%)				PRESELECTION ATC ≥ 6 (22.5%)			PRESELECTION ATC≥ 7 (18.2%)		
Combination	**Prevalence**	**AUC Panel (95% C.I.)**	**AUC Fried (95% C.I.)**	**Prevalence**	**AUC Panel**	**AUC Fried**	**Prevalence**	**AUC Panel (95% C.I.)**	**AUC Fried (95% C.I.)**
Judgment GP	**13.5%**	**0.665 (0.642-0.688)**	**0.703 (0.675-0.735)**	11.7%	0.660 (0.636-0.683)	0.685 (0.655-0.716)	11.6%	0.660 (0.636-0.683)	0.685 (0.655-0.716)
PRISMA7									
≥ 3	**13.6%**	**0.721 (0.698-0.743)**	**0.742 (0.713-0.771)**	10.7%	0.689 (0.666-0.712)	0.729 (0.699-0.759)	10.7%	0.689 (0.666-0.712)	0.729 (0.699-0.759)
≥ 4	6.6%	0.631 (0.590-0.637)	0.668 (0.636-0.699)	4.8%	0.608 (0.584-0.631)	0.648 (0.617-0.680)	4.8%	0.608 (0.584-0.631)	0.648 (0.617-0.680)
≥ 5	1.2%	0.527 (0.504-0.550)	0.552 (0.521-0.582)	1.2%	0.527 (0.504-0.550)	0.552 (0.521-0.582)	1.2%	0.527 (0.504-0.550)	0.552 (0.521-0.582)
GFI									
≥ 4	**14.7%**	**0.657 (0.633-0.678)**	0.724 (0.695-0.754)	**12.9%**	0.650 (0.627-0.673)	**0.735 (0.705-0.764)**	12.9%	0.650 (0.627-0.673)	0.735 (0.705-0.764)
≥ 5	11.8%	0.624 (0.601-0.647)	0.685 (0.655-0.715)	10.6%	0.632 (0.609-0.656)	0.692 (0.661-0.722)	10.6%	0.632 (0.609-0.656)	0.692 (0.661-0.722)
≥ 6	7.8%	0.570 (0.547-0.593)	0.602 (0.571-0.633)	6.6%	0.578 (0.555-0.601)	0.609 (0.578-0.640)	6.6%	0.578 (0.555-0.601)	0.609 (0.578-0.640)
EFS									
≥4	**7.6%**	**0.670 (0.646-0.693)**	**0.709 (0.678-0.740)**	7.6%	0.670 (0.646-0.693)	0.709 (0.678-0.740)	7.6%	0.670 (0.646-0.693)	0.709 (0.678-0.740)
≥6	3.6%	0.581 (0.557-0.604)	0.567 (0.537-0.598)	3.6%	0.581 (0.557-0.604)	0.567 (0.537-0.698)	3.6%	0.581 (0.557-0.604)	0.567 (0.537-0.598)
ISAR PC									
≥2	23.0%	0.601 (0.578-0.623)	0.648 (0.619-0.678)	16.6%	0.643 (0.621-0.666)	0.685 (0.655-0.714)	12.3%	0.672 (0.649-0.695)	0.709 (0.679-0.739)
≥3	21.9%	0.608 (0.586-0.631)	0.655 (0.625-0.684)	16.6%	0.643 (0.621-0.666)	0.685 (0.655-0.714)	**12.3%**	**0.672 (0.649-0.695)**	**0.709 (0.679-0.739)**
FI									
≥ 0.20	**13.6%**	**0.686 (0.663-0.709)**	0.704 (0.674-0.734)	13.0%	0.673 (0.650-0.696)	0.708 (0.678-0.737)	13.0%	0.673 (0.650-0.696)	0.708 (0.678-0.737)
≥ 0.25	**7.6%**	0.656 (0.633-0.680)	**0.738 (0.708-0.768)**	7.6%	0.656 (0.633-0.680)	0.738 (0.708-0.768)	7.6%	0.656 (0.633-0.680)	0.738 (0.708-0.768)
≥ 0.30	4.8%	0.608 (0.584-0.631)	0.648 (0.617-0.680)	4.8%	0.608 (0.584-0.631)	0.648 (0.617-0.680)	4.8%	0.608 (0.584-0.631)	0.648 (0.617-0.680)
≥ 0.35	3.6%	0.581 (0.557-0.604)	0.626 (0.594-0.657)	3.6%	0.581 (0.557-0.604)	0.626 (0.594-0.657)	3.6%	0.581 (0.557-0.604)	0.626 (0.594-0.657)
≥ 0.40	1.8%	0.540 (0.518-0.563)	0.578 (0.547-0.608)	1.8%	0.540 (0.518-0.563)	0.578 (0.547-0.608)	1.8%	0.540 (0.518-0.563)	0.578 (0.547-0.608)
InterRAI	**8.2%**	**0.666 (0.642-0.689)**	**0.735 (0.704-0.765)**	7.6%	0.652 (0.629-0.676)	0.709 (0.678-0.740)	7.6%	0.652 (0.629-0.676)	0.709 (0.678-0.740)
Gait speed	**10.5%**	**0.659 (0.635-0.683)**	**0.766 (0.736-0.796)**	8.8%	0.617 (0.593-0.641)	0.745 (0.715-0.776)	7.6%	0.625 (0.601-0.649)	0.752 (0.721-0.783)
Self-rated Health									
≤ 7	**14.8%**	**0.690 (0.668-0.713)**	0.754 (0.725-0.782)	13.6%	0.681 (0.658-0.704)	0.760 (0.732-0.789)	12.4%	0.689 (0.666-0.712)	**0.767 (0.738-0.795)**
≤ 6	6.0%	0.617 (0.594-0.641)	0.641 (0.610-0.673)	6.0%	0.617 (0.594-0.641)	0.641 (0.610-0.673)	6.0%	0.617 (0.594-0.641)	0.641 (0.610-0.673)
≤ 5	3.6%	0.581 (0.557-0.604)	0.597 (0.565-0.628)	3.6%	0.581 (0.557-0.604)	0.597 (0.565-0.628)	3.6%	0.581 (0.557-0.604)	0.597 (0.565-0.628)
Polypharmacy									

PRESELECTION ICPC ≥ 2 (52.5%)				PRESELECTION ICPC ≥ 3 (30.7%)			PRESELECTION ICPC ≥ 4 (17.8%)		
Combination	**Preval ence**	**AUC Panel (95% C.I.)**	**AUC Fried (95% C.I.)**	**Preval ence**	**AUC Panel**	**AUC Fried**	**Prevale nce**	**AUC Panel (95% C.I.)**	**AUC Fried (95% C.I.)**
Judgment GP	**23.9%**	**0.716 (0.694-0.737)**	**0.731 (0.704-0.758)**	13.8%	0.644 (0.621-0.667)	0.644(0.614-0.675)	9.1%	0.652 (0.629-0.676)	0.642 (0.611-0.673)
PRISMA7									
≥ 3	**18.3%**	**0.724 (0.702-0.746)**	**0.801 (0.775-0.826)**	11.2%	0.667 (0.644-0.690)	0.697 (0.667-0.728)	7.7%	0.639 (0.616-0.663)	0.688 (0.657-0.719)
≥ 4	10.7%	0.672 (0.649-0.695)	0.700 (0.670-0.731)	6.5%	0.612 (0.689-0.636)	0.609 (o.578-0.641)	4.2%	0.594 (0.571-0.618)	0.622 (0.591-0.654)
≥ 5	3%	0.533 (0.510-0.555)	0.598 (0.566-0.629)	1.8%	0.540 (0.518-0.563)	0.548 (0.518-0.579)	1.8%	0.540 (0.518-0.563)	0.548 (0.518-0.579
GFI									
≥ 4	25.9%	0.668 (0.647-0.690)	0.690 (0.662-0.718)	16.4%	0.644 (0.621-0.667)	0.656 (0.626-0.686)	10.5%	0.666 (0.643-0.689)	0.661 (0.630-0.691)
≥ 5	**18.9%**	**0.680 (0.658-0.703)**	**0.730 (0.702-0.758)**	12.9%	0.650 (0.627-0.673)	0.676 (0.646-0.707)	9.3%	0.657 (0.634-0.680)	0.667 (0.637-0.698)
≥ 6	9.6%	0.593 (0.570-0.616)	0.621 (0.590-0.652)	4.8%	0.573 (0.550-0.596)	0.561 (0.539-0.591)	3.0%	0.567 (0.544-0.590)	0.541 (0.511-0.571)
EFS									
≥4	**12.8%**	**0.685 (0.662-0.708)**	**0.764 (0.735-0.792)**	7.7%	0.652 (0.629-0.676)	0.709 (0.678-0.739)	6.4%	0.643 (0.619-0.666)	0.686 (0.655-0.718)
≥6	4.2%	0.559 (0.536-0.583)	0.620 (0.589-0.651)	3.0%	0.567 (0.544-0.590)	0.571 (0.540-0.601)	3.0%	0.567 (0.544-0.590)	0.571 (0.540-0.601)
ISAR PC									
≥2	30.3%	0.689 (0.668-0.710)	0.722 (0.714-0.767)	17.7%	0.669 (0.647-0.692)	0.678 (0.649-0.708)	9.9%	0.670 (0.647-0.693)	0.693 (0.663-0.724)
≥3	**26.9%**	**0.711 (0.690-0.732)**	**0.740 (0.696-0.748)**	15%	0.687 (0.664-0.710)	0.694 (0.664-0.723)	9.9%	0.670 (0.647-0.693)	0.693 (0.663-0.724)
FI									
≥ 0.20	**26%**	**0.759 (0.738-0.779)**	0.775 (0.751-0.800)	15.9%	0.687 (0.665-0.710)	0.689 (0.659-0.719)	10.7%	0.705 (0.682-0.728)	0.689 (0.659-0.720)
≥ 0.25	**13.4%**	0.720 (0.697-0.742)	**0.791 (0.763-0.818)**	10.4%	0.654 (0.631-0.677)	0.720 (0.690-0.750)	7.6%	0.655 (0.631-0.678)	0.706 (0.676-0.737)
≥ 0.30	6.7%	0.630 (0.607-0.654)	0.667 (0.636-0.698)	5.5%	0.604 (0.580-0.627)	0.615 (0.584-0.628)	4.3%	0.594 (0.571-0.618)	0.593 (0.562-0.628)
≥ 0.35	4.3%	0.594 (0.571-0.618)	0.622 (0.591-0.654)	3.6%	0.581 (0.557-0.604)	0.596 (0.565-0.628)	3.6%	0.581 (0.557-0.604)	0.596 (0.565-0.628)
≥ 0.40	1.8%	0.540 (0.517-0.563)	0.578 (0.547-0.608)	1.8%	0.540 (0.517-0.563)	0.578 (0.547-0.608)	1.8%	0.540 (0.517-0.563)	0.578 (0.547-0.608)
InterRAI	**13.8%**	**0.679 (0.656-0.702)**	**0.759 (0.730-0.787)**	9.7%	0.638 (0.614-0.661)	0.667 (0.636-0.698)	7.6%	0.652 (0.629-0.676)	0.680 (0.649-0.711)
Gait speed	**16.3%**	**0.727 (0.704-0.750)**	**0.883 (0.862-0.904)**	11.7%	0.678 (0.654-0.703)	0.787 (0.758-0.815)	7.5%	0.650 (0.626-0.675)	0.780 (0.750-0.810)
Self-rated Health									
≤ 7	**20%**	**0.667 (0.644-0.689)**	0.736 (0.734-0.792)	12.5%	0.647 (0.623-0.671)	0.674 (0.642-0.706)	9.4%	0.649 (0.625-0.673)	0.659 (0.627-0.692)
≤ 6	7.8%	0.630 (0.606-0.654)	**0.741 (0.707-0.774)**	4.3%	0.581 (0.557-0.605)	0.637 (0.603-0.670)	3.6%	0.567 (0.543-0.591)	0.608 (0.575-0.641)
≤ 5	4.2%	0.599 (0.575-0.623)	0.654 (0.619-0.689)	2.4%	0.609 (0.586-0.633)	0.583 (0.550-0.615)	2.4%	0.557 (0.533-0.580)	0.583 (0.550-0.615)
Polypharmacy									
≥5	**20.2%**	0.696 (0.667-0.725)	**0.696 (0.665-0.726)**	12.9%	0.621 (0.598-0.644)	0.679 (0.649-0.709)	9.9%	0.641 (0.617-0.664)	0.696 (0.665-0.726)
≥6	15%	0.696 (0.666-0.726)	0.673 (0.642-0.704)	10.5%	0.602 (0.579-0.625)	0.663 (0.632-0.694)	8.7%	0.614 (0.590-0.637)	0.673 (0.642-0.704)
≥7	12.3%	**0.711 (0.681-0.741)**	0.680 (0.649-0.711)	9.4%	0.609 (0.586-0.633)	0.669 (0.639-0.700)	7.6%	0.621 (0.598-0.645)	0.680 (0.649-0.711)

PRESELECTION GP (28.6%)			
Combination	**Prevalence**	**AUC Panel (95% C.I.)**	**AUC Fried (95% C.I.)**
Judgment GP			
PRISMA7			
≥ 3	**15.5%**	**0.727 (0.705-0.749)**	**0.732 (0.703-0.760)**
≥ 4	10.1%	0.658 (0.635-0.682)	0.674 (0.644-0.705)
≥ 5	2.4%	0.519 (0.497-0.542)	0.572 (0.541-0.603)
GFI			
≥ 4	17.7%	0.690 (0.667-0.712)	0.681 (0.651-0.710)
≥ 5	**12.4%**	**0.707 (0.685-0.730)**	**0.711 (0.681-0.741)**
≥ 6	7.2%	0.627 (0.603-0.650)	0.664 (0.633-0.695)
EFS			
≥4	**12.3%**	**0.724 (0.702-0.747)**	**0.738 (0.709-0.768)**
≥6	4.2%	0.560 (0.537-0.583)	0.591 (0.560-0.622)
ISAR PC			
≥2	22.8%	0.723 (0.701-0.744)	0.679 (0.650-0.708)
≥3	**21%**	**0.734 (0.713-0.756)**	**0.689 (0.660-0.718)**
FI			
≥ 0.20	**18.2%**	**0.772 (0.751-0.793)**	0.763 (0.736-0.790)
≥ 0.25	**9.9%**	0.722 (0.699-0.754)	**0.754 (0.725-0.783)**
≥ 0.30	6.0%	0.635 (0.611-0.658)	0.671 (0.639-0.702)
≥ 0.35	4.2%	0.594 (0.671-0.618)	0.622 (0.591-0.654)
≥ 0.40	1.8%	0.540 (0.518-0.563)	0.578 (0.547-0.608)
InterRAI	**11.7%**	**0.711 (0.688-0.733)**	**0.771 (0.742-0.799)**
Gait speed	**12.6%**	**0.682 (0.658-0.707)**	**0.801 (0.773-0.830)**
Self-rated Health	**13.3%**	**0.734 (0.704-0.765)**	**0.668 (0.645-0.691)**
≤ 7	6.8%	0.655 (0.622-0.688)	0.630 (0.607-0.654)
≤ 6	3.7%	0.608 (0.575-0.641)	0.581 (0.557-0.604)
≤ 5			
Polypharmacy	13.3%	**0.705 (0.675-0.735)**	**0.665 (0.642-0.688)**
≥5	11.7%	0.685 (0.655-0.716)	0.660 (0.636-0.683)
≥6	11.7%	0.685 (0.655-0.716)	0.660 (0.636-0.683)
≥7			

*Highest AUC-values are highlighted for every preselection method.
